# The distinctive profile of risk factors of nasopharyngeal carcinoma in comparison with other head and neck cancer types

**DOI:** 10.1186/1471-2458-8-400

**Published:** 2008-12-05

**Authors:** AS Abdulamir, RR Hafidh, N Abdulmuhaimen, F Abubakar, KA Abbas

**Affiliations:** 1 Microbiology Research Department, University Putra Malaysia, 43400, UPM, Serdang, Malaysia; 2 Institute of Bioscience, University Putra Malaysia, 43400, UPM, Serdang, Selangor Darul Ehsan, Malaysia; 3 Medical Microbiology Department, College of Medicine, Alnahrain University, Iraq; 4 Faculty of Food Science and Technology, University Putra Malaysia, 43400, UPM, Serdang, Malaysia

## Abstract

**Background:**

Nasopharyngeal carcinoma (NPC) and other head and neck cancer (HNCA) types show a great epidemiological variation in different regions of the world. NPC has multifactorial etiology and many interacting risk factors are involved in NPC development mainly Epstein Barr virus (EBV). There is a need to scrutinize the complicated network of risk factors affecting NPC and how far they are different from that of other HNCA types.

**Methods:**

122 HNCA patients and 100 control subjects were studied in the region of the Middle East. Three types of HNCA were involved in our study, NPC, carcinoma of larynx (CL), and hypopharyngeal carcinoma (HPC). The risk factors studied were the level of EBV serum IgG and IgA antibodies measured by ELISA, age, sex, smoking, alcohol intake, histology, and family history of the disease.

**Results:**

EBV serum level of IgG and IgA antibodies was higher in NPC than CL, HPC, and control groups (p < 0.01). NPC was associated with lymphoepithelioma (LE) tumors, males, regular alcohol intake, and regular smoking while CL and HPC were not (p < 0.05). CL and HPC were associated with SCC tumors (p < 0.05). Furthermore, NPC, unlike CL and HPC groups, was not affected by the positive family history of HNCA (p > 0.05). The serum levels of EBV IgG and IgA antibodies were higher in LE tumors, regular smokers, younger patients, and negative family history groups of NPC patients than SCC tumors, non-regular smokers, older patients and positive family history groups respectively (p < 0.05) while this was not found in the regular alcoholics (p > 0.05).

**Conclusion:**

It was concluded that risk factors of NPC deviate much from that of other HNCA. EBV, smoking, alcohol intake, LE tumors, male patient, and age > 54 years were hot risk factors of NPC while SCC and positive family history of the disease were not. Earlier incidence, smoking, LE tumors, and negative family history of the disease in NPC patients were associated much clearly with EBV. It is proposed that determining the correct risk factors of NPC is vital in assigning the correct risk groups of NPC which helps the early detection and screening of NPC.

## Background

Nasopharyngeal carcinoma (NPC) was shown to be distinct from other head and neck cancer (HNCA) types in terms of histopathological spectrum, and geographical distribution [1]. In the Western world, NPC is an uncommon type of tumor. In the USA, NPC represents less than 1% of all cancers. The annual incidence in the USA and Europe varies between 0.22 and 0.5 per 100 000 population [2,3]. In contrast, NPC is widely prevalent in South East Asia, the Middle East, and North Africa where higher incidence of NPC has been reported than other parts of the world [4,5]. However, China and Southeast Asian countries have been considered as the highest incidence regions for NPC in the world where incidence could reach 20 to 50 per 100 000 individuals [4-8]. Nevertheless, high NPC incidence rate, 92/100,000 was found in some parts of the Middle East in 2002–2003 [9]. These figures are surprisingly higher than that in China and South East Asia making the Middle East region as one of the highly endemic regions for NPC. Unfortunately, the high incidence of NPC in this region has long been underestimated. Moreover, the risk factors of NPC development have not been studied thoroughly in this region of the world.

NPC is highly metastatic and invasive malignant tumor. Approximately 90% of NPC patients show malignant cervical lymph nodes [10]. Epstein-Barr virus (EBV) is considered as one of the main etiological factors of NPC, which is an oncogenic herpesvirus associated with a variety of malignancies in T cells, B cells, and epithelial cells [11]. EBV establishes a life-long persistent infection in over 90% of the human adult population worldwide [12]. EBV has been linked to the development of a variety of human malignancies of lymphoid and epithelial origin, including Burkitts lymphoma, Hodgkin's lymphoma, and NPC [13].

In this study, NPC patients were the core group for the comparison with other HNCA patients in terms of the studied risk factors namely, age, sex, staging of the disease, histology of the tumors, smoking, alcohol intake, family history of the disease, and EBV serology. The aim of this study was to evaluate precisely the essential risk factors and assign correctly the high risk groups of NPC and other HNCA types in the population of the Middle East where a remarkable shortage of research has been present.

## Methods

### The population of the study

One hundred twenty two HNCA patients were selected without any bias to any type during the period between January 2006 to January 2008 in the University hospital of the Medical College of Alnahrain University and Radiotherapy Reference Center in Baghdad, Iraq, and from Alhussein Hospital in Amman, Jordan. The samples were processed and the study was totally conducted in University Putra Malaysia (UPM). HNCA patients were involved after the diagnosis was established. Primary HNCA cases were selected rather than secondary or recurrent cases. HNCA patients were 42 NPC patients, 66 carcinoma of larynx (CL), and 14 hypopharyngeal carcinoma (HPC) patients. HNCA patients and control subjects in Iraq and Jordan were native Middle Eastern people of Arabic ethnicity. Twenty patients of other HNCA types that were of small sample size, less than 6 patients, were neglected for statistical purposes such as tongue, retromalar, tonsillar, epiglottic and post-pharyngeal carcinomas. The age of HNCA patients ranged between 16 to 74 years old, median, 53 years and mean 51.8 years. The median age of NPC patients was 54.4 years and the mean age was 54 years. For HPC patients, the median age was 62.5 years and the mean age was 62.16 years. For CL patients, the median age was 50.5 years and the mean age was 50.81 years. HNCA patients were 89 (72.9%) males and 33 (27%) females at male:female ratio, 2.6:1. NPC patients were 9 (21.4%) females and 33 (78.5%) males revealing male predominance at sex ratio, 3.6:1. HPC patients were 12 (85.7%) females and 2 (14.3%) males with females predominance at sex ratio 1:6. CL patients were 6 (9%) females and 60 (91%) males with clear male predominance at sex ratio 10:1. HNCA staging was based on TNM system by which HNCA patients were classified into 4 stages, depending on T (the extent of primary tumor), N (regional LN spread) and M (distant metastasis) [14]. Accordingly, the studied HNCA patients were diagnosed at different stages of the disease, 68% at stage IV, 21% at stage III, 7% at stage II, and 4% at stage I.

One hundred age- and sex- matched control subjects were selected. Control subjects were those who attended hospitals for minor traumatic therapy. They showed normal blood and biochemical laboratory tests, namely differential blood count, hemoglobin level, total serum proteins, liver function tests, erythrocytes sedimentation rate, kidney function tests, and C-reactive proteins as well as were medically tested by a specialist and found free of HNCA or any other medical illness.

### The survey data and sampling

Direct interview was done with HNCA patients. The data sheet for survey encompassed name, age, sex, smoking, the number of smoked cigarettes per day, how many years the smoking habit has been, alcohol consumption, level and duration of alcohol consumption, the type of alcohol drink, the family history of HNCA (parents, brothers/sisters, cousins and children). Moreover, the past and recent medical records and the reports of histopathology of the involved patients were reviewed. The cutoff line of regular smoking (smoking > 10 cigarettes per day more than 10 years) was used in this study. In terms of Brinkman index, number of cigarettes smoked per day multiplied by number of years of smoking, this cutoff line is equal to 100. The cutoff line for regular alcohol consumption is equal to or more than 2 glasses per day (about 400–500 mL/day) of drinks with alcohol concentration more than 12% for 10 years and more. This is equal to ethanol consumption > 210 g/week for more than 10 years. Above this cutoff value, individuals were considered regular alcoholics [15].

Sera were sampled from HNCA patients before the beginning of chemotherapy or surgery and from control subjects after excluding any HNCA and other medical illness. Sera were used to estimate the serum level of EBV IgG and IgA antibodies via ELISA. After surgery, paraffin embedded sections were retrieved from the excisional biopsies of HNCA primary tumors. The histopathological examination and classification was carried out by a histologist. The histopathology of HNCA tumor sections was assessed and classified for purposes of comparison into two main groups; well-differentiated squamous cell carcinoma (SCC) and undifferentiated lymphoepithelioma (LE). Written consents were granted by HNCA patients and control subjects. The procedures of specimens sampling and patient interviewing were done according to the guidelines of Helsinki declaration for the ethics of the biomedical research. Official permission was granted by the regional Ethics committee of medical research in Baghdad and Amman clinical institutes.

### ELISA for Epstein Barr virus serum antigens

EBV viral capsid antigen (VCA) (Wellcome) was used for coating the solid phase of 96 wells microtiter plates. The antigen was diluted 1:10 in carbonate-bicarbonate coating buffer (1.59 g/L carbonate & 2.93 g/L bicarbonate) (Sigma). After a series of standardization steps, 50 μl/well of the EBV antigen in coating buffer were added for overnight at 4°C. Next day, the remaining fluid was aspirated and wells were washed twice with washing buffer (PBS from Sigma, 1% BSA w/v from Merck, 0.05% v/v Tween 20 from Merck). Blocking buffer (PBS, 1% BSA w/v) was then added for 1 hour. After washing step, 50 ul/well of non-diluted serum of HNCA patients and control subjects were added in duplicates. The ELISA plate was incubated for 2 hours at 37°C. Two sets of ELISA were pursued, the first for detecting EBV serum IgG antibodies and the second for detecting EBV serum IgA antibodies. After washing step, 50 ul/well of horseradish peroxidase-labeled rabbit anti-human IgG and IgA antibodies were added at 1:40 and 1:100 dilution respectively of original concentration 2 mg/mL for anti-human IgG and 3 mg/mL for anti-human IgA (ICN immunologicals). After washing step, 50 ul of the chromogen-substrate, OPD-H2O2, (Sanofi diagnostics) were added and incubated for 15 minutes for the development of the enzymatic reaction of horseradish peroxidase. ELISA readings in terms of optical density (OD) were measured immediately by microplate reader A-1764A (Beckman) at wavelength of 492 nm [16]. Duplicate wells of negative control, distilled water instead of serum, and duplicate wells of positive control solutions (Wellcome) at standardized working concentration, 10 ug/mL of EBV VCA antigen, were used at each run of ELISA. Since ELISA was used for measuring the burden of EBV serum IgG and IgA antibodies for comparative purposes among the studied risk factor groups of HNCA patients, no cutoff value was needed for demarcating sera into seronegative or seropositive nor calibration curve was needed to measure the real concentration of antigens.

### Statistical analysis

The statistical analysis was preformed using software Statistica version 6.1.478.0 and MS Excel 2000. ELISA OD values were presented as mean ± 2 standard error. After confirming the normal distribution pattern of ELISA values by Kolmogorov and Smirnov tests, parametric multivariate student t-tests were used to evaluate the significance of difference among the mean ELISA OD readings for EBV serum IgG and IgA antibodies. For qualitative categorized data (number of subjects showing certain event), the significance of association was measured by applying Chi square test of independence for contingency tables and corrected by Fishers' Exact test when needed. The histograms used for ELISA OD mean values were shown with error bars and p values of significant difference, while histograms of observational data, number of subjects and their parentages, were not shown with p values and error bars as chi square test for independence measures the significant association of variables' frequency rather than the significant difference of the variables' values.

## Results

### Serum level of EBV IgG and IgA antibodies

**Figure 1:**
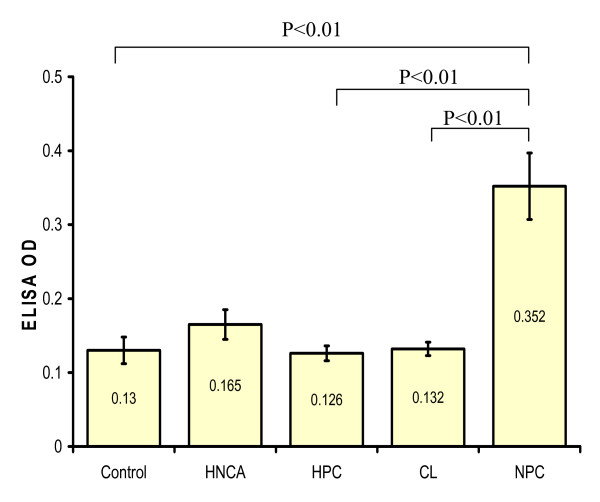
**This histogram shows the difference in the mean serum level of EBV IgG antibodies measured in ELISA OD in HNCA as one group, NPC, HPC, and CL patients as well as control subjects**. The mean serum level of EBV IgG antibodies in NPC patients was significantly higher than in CL, HPC, HNCA and control groups.

**Figure 2:**
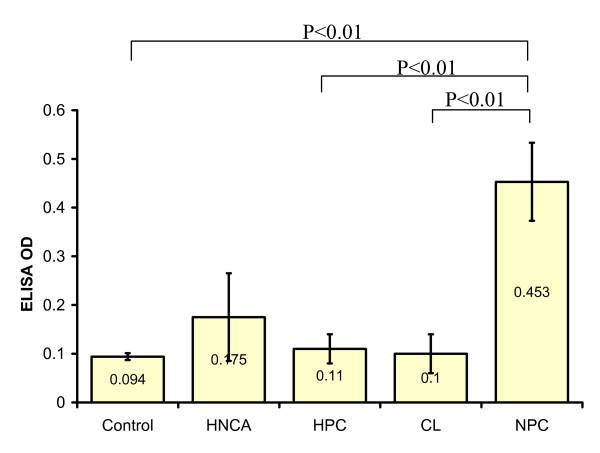
**This histogram shows the difference in the mean serum level of EBV IgA antibodies measured in ELISA OD in HNCA as one group, NPC, HPC, and CL patients as well as control subjects**. The mean serum level of EBV IgA antibodies in NPC patients was significantly higher than in CL, HPC, HNCA and control groups.

ELISA was used to assess the serum level of EBV IgG and IgA antibodies among HNCA patients in comparison with that of control subjects. The serum IgG and IgA level in HNCA patients, 0.165 ± 0.02 and 0.175 ± 0.09 respectively, were not significantly higher than in control subjects, 0.13 ± 0.018 and 0.094 ± 0.007 respectively (p > 0.05). Similarly, EBV serum level of IgG and IgA antibodies in both CL, 0.132 ± 0.009 and 0.1 ± 0.04 respectively, and HPC, 0.126 ± 0.01 and 0.11 ± 0.03 respectively, patients was not significantly higher than in control group (p > 0.05). On the other hand NPC was the only group that showed a significantly higher serum level of EBV IgG and IgA antibodies, 0.352 ± 0.045 and 0.453 ± 0.08 respectively, than control group (p < 0.01). Furthermore, NPC group showed far highest IgG and IgA levels in comparison with CL and HPC (p < 0.001) (Table 1, Fig. 1 and Fig. 2). This indicated a strong association between EBV and NPC represented by very high levels of serum EBV IgG and IgA antibodies. Therefore, EBV seems to play an essential role as biological risk factor for the development of NPC rather than other types of HNCA. On the other hand, there was no association between EBV serum IgG and IgA level and staging of the disease (p > 0.05).

**Table 1:** The mean ± 2 standard error of ELISA OD readings of EBV serum IgG and IgA antibodies in control, HNCA, NPC, CL, and HPC groups

Group	Mean ELISA OD EBV serum IgG	Mean ELISA OD EBV serum IgA
Control	0.13 ± 0.018	0.094 ± 0.007
HNCA	0.165 ± 0.02	0.175 ± 0.09
NPC	0.352 ± 0.045	0.453 ± 0.08
CL	0.132 ± 0.009	0.1 ± 0.04
HPC	0.126 ± 0.01	0.11 ± 0.03

### The histological characteristics of HNCA patients

**Figure 3:**
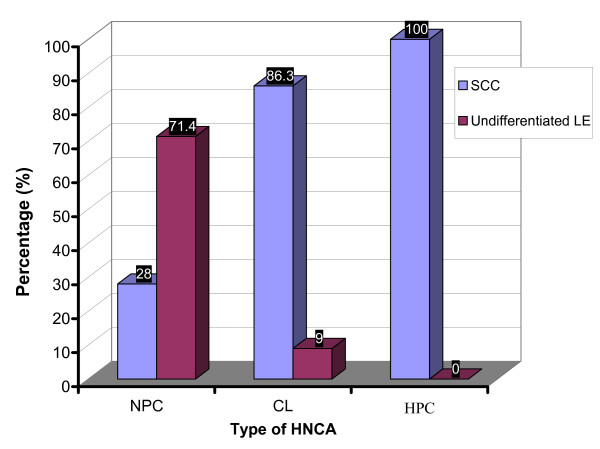
**This histogram shows the percentage of patients with tissue sections of SCC and LE tumors in each group of HNCA patients**. It was shown that LE tumors were associated with NPC group while SCC tumors were associated with CL and HPC groups.

**Figure 4:**
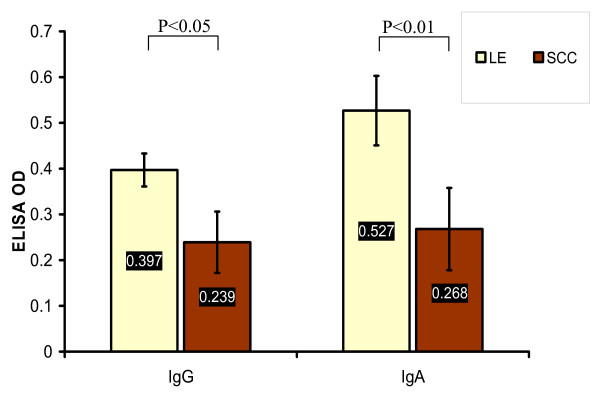
**This histogram shows the significant higher mean values of EBV serum IgG and IgA antibodies, measured in ELISA OD, in NPC patients with LE tumors than in NPC patients with SCC tumors**.

SCC was dominant in HNCA patients except for NPC patients who elicited a different histological profile in that large proportion of highly undifferentiated carcinoma, namely LE, was found. It was 12 (28.5%) of NPC patients whose histopathological paraffin-embedded sections showed SCC versus 30 (71.4%) LE. In CL group, 57 (86.3%) patients whose histopathological paraffin-embedded sections were SCC, 6 (9%) undifferentiated LE and 3 (4.5%) adenocarcinoma. In HPC group, the histopathological paraffin-embedded sections of all patients were SCC (Table 2 and Fig. 3). The chi square test of independence showed that NPC group was associated with LE versus CL and HPC groups were associated with SCC (p < 0.05). In addition, the serum level of EBV IgG and IgA antibodies proved to be associated with tumor histology. Mean ELISA readings of EBV serum IgG and IgA antibodies in NPC patients with LE, 0.397 ± 0.036 and 0.527 ± 0.076 respectively, were much higher than that of NPC patients with SCC, 0.239 ± 0.067 (*p *< 0.05) and 0.268 ± 0.09 respectively (p < 0.01) (Table 3 and Fig. 4). This provided evidence that EBV is associated clearly with the undifferentiated NPC of LE histology rather than differentiated NPC of SCC histology. And this association can be estimated well by monitoring EBV serum antibodies especially IgA antibodies which reflected this association more obviously than IgG antibodies. Although 9% of CL patients were of LE tumors, no similar association was found between EBV serum IgG and IgA level and CL tumor histology. The serum level of IgG, 0.135 ± 0.01, and IgA, 0.11 ± 0.032, in CL patients with LE tumors was not different from that in CL patients with SCC tumors, 0.131 ± 0.008 and 0.093 ± 0.044 respectively (p > 0.05) (Table 3). This provided another clue on the particular association of EBV with LE histology of NPC rather than other types of HNCA.

**Table 2:** The number and percentage of patients/subjects with the studied observations of this study in control, NPC, CL, and HPC groups

**Variable**	**NPC N(%)**	**CL N(%)**	**HPC N(%)**	**Control N(%)**
Histopathology of tumors:				
SCC	12 (28.5)	57 (86.3)	14 (100)	-
LE	30 (71.4)	6 (9%)	0	-
adenocarcinoma	0	3 (4.5)	0	-
Regular smokers	36 (85.7)	47 (71.2)	4 (28.6)	25 (36.7)
Regular alcohol consumers	18 (42.8)	10 (15.1)	3 (21.4)	9 (13.3)
SCC in the regular alcohol consumers	9 (50)	-	-	-
SCC in the non-regular alcohol consumers	3 (12.5)	-	-	-
HNCA-positive family history	6 (14.3)	33 (50%)	9 (64.3)	-

**Table 3:** The mean ± 2 standard error of ELISA OD readings of EBV serum IgG and IgA antibodies in different subgroups of NPC and CL patients

**Group**	**Mean ELISA OD EBV serum IgG**	**Mean ELISA OD EBV serum IgA**
NPC patients with LE	0.397 ± 0.036	0.527 ± 0.076
NPC patients with SCC	0.239 ± 0.067	0.268 ± 0.09
		
CL patients with LE	0.135 ± 0.01	0.11 ± 0.032
CL patients with SCC	0.131 ± 0.008	0.093 ± 0.044
		
Below-median age group of NPC	0.314 ± 0.039	0.486 ± 0.073
Above-median age group of NPC	0.327 ± 0.047	0.3 ± 0.082
		
Regular smokers in NPC	0.379 ± 0.043	0.493 ± 0.084
Non- regular smokers in NPC	0.19 ± 0.057	0.213 ± 0.056
		
Regular alcohol consumers in NPC	0.325 ± 0.05	0.466 ± 0.072
Non-regular alcohol consumers in NPC	0.372 ± 0.041	0.445 ± 0.086
		
HNCA-positive family history group of NPC	0.2 ± 0.065	0.177 ± 0.1
HNCA-negative family history group of NPC	0.377 ± 0.041	0.499 ± 0.076

### Age and Sex factors in HNCA patients

**Figure 5:**
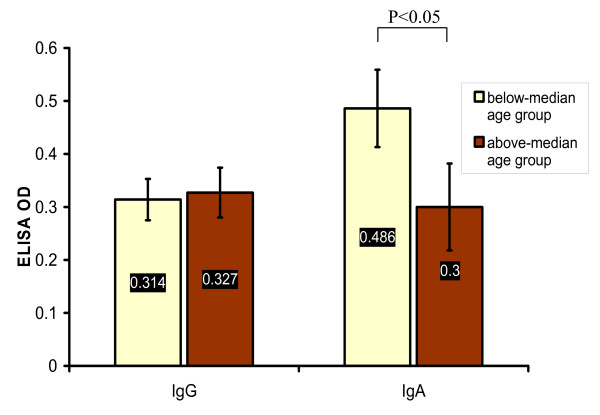
**This histogram shows the significant higher mean values of EBV serum IgA but not IgG antibodies, measured in ELISA OD, in NPC patients with age below the median cutoff value (54.4 years) than in NPC patients with age above the median cutoff value (54.4 years)**.

There was no insignificant difference among the mean age of NPC, HPC and CL patients, 54 ± 3.6, 62.16 ± 4.1, and 50.81 ± 5.3 respectively providing evidence that age of HNCA patients was not distinct for each type of HNCA (p > 0.05). In attempt to disclose any possible association of NPC patients age with EBV serum IgG and IgA antibodies, the median age of NPC patients, 54.4 years, was used to group NPC patients into above-median age group and below-median age group. The serum level of EBV IgA, 0.486 ± 0.073, in below-median age group was higher than that, 0.3 ± 0.082, in above-median age group (p < 0.05) but the serum level of IgG, 0.314 ± 0.039, in below-median age group was not higher than that, 0.327 ± 0.047, in above-median age group (p > 0.05) (Table 3 and Fig. 5). Age of NPC, CL, and HPC patients was not associated with the staging of the disease (p > 0.05).

It was shown that sex ratio in HNCA groups was significantly different from each other (p < 0.01) in that HPC group showed the lowest male:female ratio, 1:6, then NPC, 3.6:1 and the highest ratio was found in CL, 10:1. On the other hand, sex of NPC patients did not show a significant association with EBV serum antibodies (p > 0.05) like that shown in the age of NPC patients. There was no association between sex ratio and age of patients and staging of the disease (p > 0.05).

### Smoking

**Figure 6:**
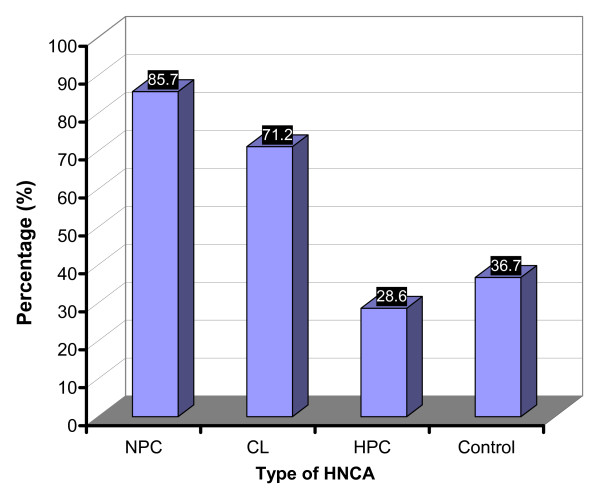
**This histogram shows the percentage of the regular smokers in the studied groups of HNCA as well as in the control subjects**. NPC and CL groups were associated with the regular smoking habit when compared to the control and HPC groups.

**Figure 7:**
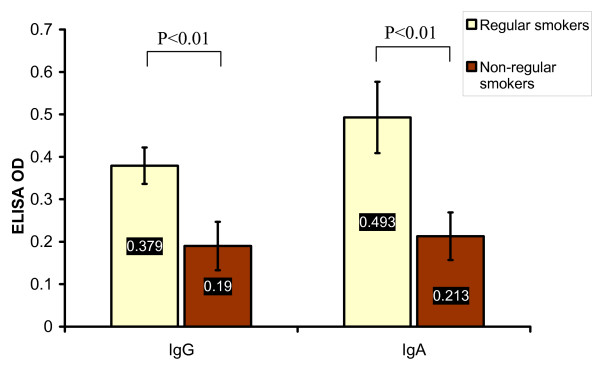
**This histogram shows the significant higher mean values of EBV serum IgG and IgA antibodies, measured in ELISA OD, in the regular smokers group of NPC patients than in the non-regular smokers group of NPC patients**.

It was found that 81 (66.3%) of HNCA patients were regular smokers while only 25 (36.7%) of controls were regular smokers (p < 0.01). It was also found that 36 (85.7%) of NPC patients, 4 (28.6%) of HPC patients and 47 (71.2%) of CL patients were regular smokers (Table 2 and Fig. 6). NPC and CL groups were both remarkably associated with high percentage of regular smokers in comparison with HPC patients and control subjects (p < 0.01). Interestingly, there was a significant association between the serum level of EBV IgG and IgA antibodies and smoking. The mean ELISA reading of EBV IgG and IgA antibodies in the regular smokers group of NPC patients, 0.379 ± 0.043 and 0.493 ± 0.084 respectively, was much higher than that of the non-regular smokers group, 0.19 ± 0.057 and 0.213 ± 0.056 respectively (p < 0.01) (Table 3 and Fig. 7). This indicated that smoking was associated with EBV and the regular smokers group of NPC patients showed higher serum EBV IgG and IgA antibodies than non-smokers which highlighted a probable link between smoking and NPC via certain role of EBV. However, smoking habit in NPC group was not associated with histology, age, staging of the disease, or sex (p > 0.05).

### Alcohol consumption

**Figure 8:**
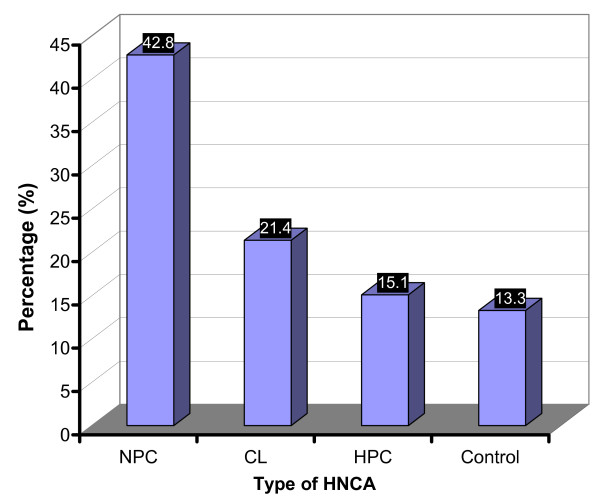
**This histogram shows the percentage of the regular alcohol consumers in the studied groups of HNCA as well as in the control subjects**. NPC was the only group associated with alcohol intake when compared to CL, HPC, and control groups.

**Figure 9:**
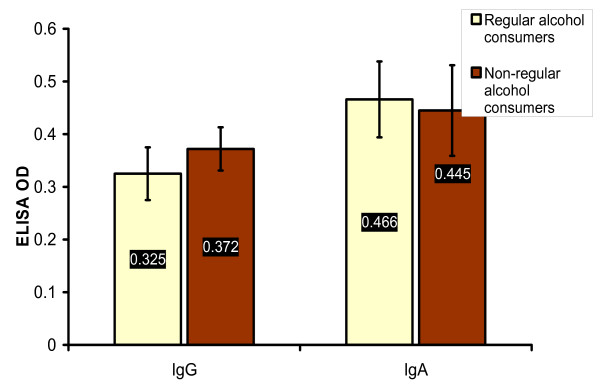
**This histogram shows that there was no significant difference in the mean values of EBV serum IgG and IgA antibodies, measured in ELISA OD, between the regular alcohol consumers group and the non-regular alcohol consumers group of NPC patients**.

Twenty eight (23%) of HNCA patients and 9 (13.3%) of control group were regular alcohol consumers (p > 0.05). The percentage of regular alcohol consumers in NPC was 18 (42.8%), in HPC 3 (21.4%) and in CL 10 (15.1%) (Table 2 and Fig. 8). Accordingly, the percentage of the regular alcohol consumers in NPC patients was very high if compared with CL, HPC and control group which indicated that NPC was significantly associated with regular alcohol intake (p < 0.01) while other types of HNCA were not. Therefore, regular alcohol consumption might be one of the remarkable risk factor for developing NPC disease. Unlike smoking, alcohol risk factor might be more interesting in the epidemiology of NPC as other HNCA groups did not show any similar association with alcohol intake. On the other hand, there was no significant association between the serum level of EBV IgG and IgA antibodies and the regular alcohol intake in NPC group. The mean ELISA readings of EBV serum IgG and IgA in the regular alcohol consumers of NPC group, 0.325 ± 0.05 and 0.466 ± 0.072 respectively, were not different from that in the non-regular alcohol consumers group, 0.372 ± 0.041 and 0.445 ± 0.086 respectively (p > 0.05), (Table 3 and Fig. 9). It was also shown that the percentage of NPC patients with SCC in the regular alcohol consumers, 9 (50%), was higher than that in the non-regular alcohol consumers, 3 (12.5%) which indicated a significant association of SCC tumors of NPC with alcohol intake (p < 0.05) (Table 2). Since SCC histology was associated with much lower levels of EBV serum IgG and IgA, the link between SCC and alcohol intake might give a clue on different mechanisms/risk factors for NPC development where EBV does not play an important role as risk factor like in LE histology. However, alcohol intake in NPC group was not associated with smoking, age, staging of the disease, or sex (p > 0.05).

### Positive family history of the disease

**Figure 10:**
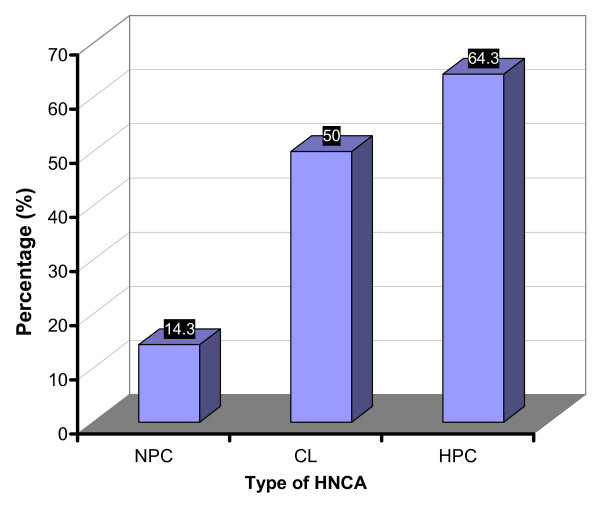
**This histogram shows the percentage of HNCA patients with positive family history of the disease**. CL and HPC groups, but not NPC group, were associated with positive family history of the disease. Therefore, the majority of NPC patients, unlike CL and HPC patients, were of negative family history of the disease.

There were 48 HNCA patients (39.3%) who showed positive family history of HNCA, i.e. a history of HNCA within the first 3 degree relatives, namely parents, brothers/sisters and cousins. It was shown that 6 (14.3%) of NPC patients revealed positive family history. On the other hand, HPC and CL patients revealed 9 (64.3%) and 33 (50%) patients respectively with positive family history (Table 2 and Fig. 10). The lowest percentage of patients with HNCA-positive family history was in NPC group which was associated significantly with the negative HNCA family history group (p < 0.05) while CL and HPC were associated significantly with positive family history group (p < 0.05), This points out to the notion that positive family history of the disease is not one of the effective risk factors for NPC development. This implies to the presence of more effective risk factors of NPC than the familial inheritance of the disease.

**Figure 11:**
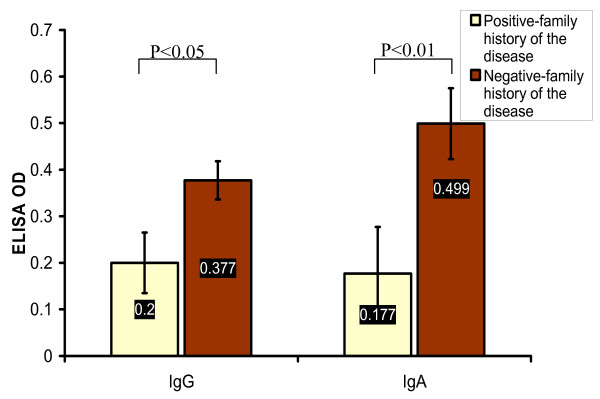
**This histogram shows the significant higher mean values of EBV serum IgG and IgA antibodies, measured in ELISA OD, in NPC patients with negative family history of the disease than in NPC patients with positive family history of the disease**.

One of the interesting results, the HNCA-positive family history group of NPC patients revealed far lower level of EBV IgG and IgA antibodies, 0.2 ± 0.065 and 0.177 ± 0.1 respectively, than in the HNCA-negative family history group, 0.377 ± 0.041 (p < 0.05) and 0.499 ± 0.076 respectively (p < 0.01), (Table 3 and Fig. 11) rendering the HNCA-negative family history group of NPC the most affected by EBV infection and vice versa. There was no association found between the HNCA-family history and histology, sex, age, staging of the disease, smoking, and alcohol intake (p > 0.05).

## Discussion

This study explored the complicated network of risk factors of NPC and other types of HNCA in one of the highly NPC-endemic regions in the world, the Middle East. The role of EBV as a risk factor of NPC was the pivotal line of research that all other risk factors were linked to. The strategy of this research was comparing the studied risk factors in; HNCA patients as one group versus control subjects and different HNCA groups versus control subjects and with each other. Moreover, it was aimed at comparing EBV serum level of IgG and IgA antibodies in different subgroups of NPC patients.

NPC patients showed the highest level of serum EBV IgG and IgA antibodies when compared to the studied HNCA groups and control group. This is in agreement with many published reports that explored the association between EBV and HNCA in general and NPC in particular [17-20]. Elevated antibody titers to EBV antigens, e.g. early antigens, viral capsid antigens are frequently found in NPC patients and have been considered as important markers in the diagnosis and prognosis of these EBV-associated diseases [17,18]. It's a special feature of NPC that patients sustain high levels of a broad spectrum of EBV antibodies especially those for IgA and IgG subtypes directly against both latent and lytic viral antigens [19,20]. Accordingly, this study showed that EBV association with NPC in the Middle East is congruous with other NPC endemic regions and provide evidence that EBV is one of the most influential risk factors for the development of NPC in this region.

There was no age distinction for each type of HNCA. The mean age of the studied HNCA groups was higher than 50 years. This revealed that age of more than 50 years could be considered as a risk factor for HNCA development. Interestingly, higher level of EBV serum IgA but not IgG was found in NPC patients who were younger than the median age cutoff line, 54.4 years. This finding showed that older NPC patients were affected less by EBV as a risk factor of NPC development. This might give a clue that the high burden of EBV infection could accelerate the occurrence of NPC in the population and this is supported by a previous report [4] stating that the mean age of NPC patients in South East Asia is lower than in Western countries. This might be explained by the context of our results that EBV is a leading risk factor in high endemic regions of NPC like South East Asia and the Middle East which might accelerate NPC incidence making the mean age of NPC patients in high endemic regions less than that in western countries where EBV is less frequently associated with NPC. However, it is not clear why EBV serum IgG antibodies were not associated with younger NPC patients like IgA antibodies. One of the possible explanations, the older NPC patients might undergo severe immune suppression which affects the IgA antibodies more than the long-lasting IgG antibodies. However, this might need further investigation in a separate study to focus on this point of the research. Nevertheless, because EBV serum IgA antibodies are more specific than IgG antibodies in respect to NPC development, the association of EBV serum IgA antibodies with younger NPC patients was an interesting finding.

Male: female ratio in HNCA patients was 2.6:1 which reflected a male predominance over females. Many reports have confirmed this outcome and revealed that under 45 years of age the incidence in men was double that in women, with the increasing age the rate of incidence increase accelerated more in men than in women [21,22]. HNCA groups showed different sex ratios. The male predominance in CL and NPC belongs largely to the nature of the risk factors in the Middle East society which affects males more than females. Men are more apt to be exposed to different risk factors due to the outdoor nature of work that most females in this region are not actively involved in. Therefore, most females, especially in the rural areas do house keeping jobs where less chances of exposure to pollution, occupational factors, passive smoking in the public regions, stress-induced immune suppression that help trigger EBV reactivation. Nevertheless, most HPC patients were females. It is worth mentioning that there was no previous report revealed female predominance in HPC patients like what found in this study. This needs further investigation to elucidate the reasons of this peculiar sex ratio in HPC patients in this region of the world.

The histopathology of NPC patients in this study was extensively different from that of other HNCA patients. The highly undifferentiated carcinoma, namely LE was dominant in NPC patients rather than SCC which was dominant in other HNCA groups. This attracts attention to the unique profile of risk/triggering factors of NPC which lead to different profiles of histopathology. In addition, the serum level of EBV IgG and IgA antibodies was significantly higher in LE group than SCC group of NPC patients confirming the principal role of EBV in the etiology of NPC especially the undifferentiated LE type of NPC. There is a study [22] stated that EBV is associated virtually with all cases of undifferentiated NPC, lymphoepithelioma, and has been classified as group I carcinogen. Therefore, the exclusive high percentage of LE tumors in NPC patients together with the unusual high levels of EBV serum IgG and IgA in NPC versus other HNCA groups and in LE group versus SCC group of NPC patients have led to the suggestion that EBV in the Middle East, or might be in any other high endemic region, plays a central role in the NPC development favoring LE histology on other types of HNCA histology.

In this study, smoking proved to be an important risk factor for the development of HNCA. The number of regular smokers among HNCA patients was much higher than in control group. Such results have been recorded in other parts of the world. A prospective study in the USA showed that heavy smokers die due to carcinoma of larynx 32 folds than age-adjusted non-smokers, and of oral cavity cancer, more than 24 folds [23]. Nevertheless, this report stated a bit different results of our study which revealed that the rank of the highest numbers of regular smoker was in NPC, CL, and then HPC patients. Therefore, smoking seems much more effective in the etiology of NPC in the Middle East patients than in the patients of other parts of the world. In addition, the EBV serum level of both IgG and IgA antibodies was significantly higher in the regular smokers group of NPC than in the non-regular smoker group. This referred to a possible link between smoking and EBV in respect to NPC etiology or propagation. These findings together indicate that NPC is largely affected by smoking habit which is associated significantly with higher EBV serum antibodies and this point out to the possibility that cigarette smoke components are one of the initiative factors for EBV reactivation within nasopharyngeal mucosa. This needs further investigation to dig deep. Once the relationship between EBV-induced NPC and smoking could be confirmed, this will open new measures for NPC epidemiology and prevention.

Unlike smoking habit, the percentage of regular alcohol consumers in HNCA patients was not significantly higher than in control group, except for NPC patients who showed a remarkable higher percentage of regular alcohol consumers than control subjects and other HNCA patients. This provided evidence that alcohol intake could acts as one of the important risk factors of NPC development. And this affection was not seen in other HNCA types making alcohol intake of particular significance to NPC patients. This finding is supported by a previous report showed that excessive alcohol consumption increases the risk of incurring cancers of oral cavity, pharynx and to a lower extent larynx [24]. Anyhow, our study did not reveal a significant association of alcohol intake with CL as shown by [24]. Unlike smoking, this study revealed no association between alcohol intake and EBV in NPC patients which might indicate that alcohol intake is an EBV-independent risk factor for the development of NPC. Hence, another factor might be associated with alcohol intake in respect to NPC development rather than EBV. It was found that the dominant histology in the regular alcohol consumers group of NPC was SCC while in the non-regular alcohol consumers group was LE. This finding gave a clue that alcohol could affect NPC development away from EBV effect which was significantly related to LE histology. Accordingly, it was proposed that alcohol intake might be the most influential risk factor of SCC tumors but not LE tumors of NPC. In other words, alcohol intake might be the most important risk factor of NPC development out of the indirect effect of EBV infection.

Cancer is a disease of multifactorial etiology involving environmental and genetic factors. The genetic susceptibility is best measured by the family history of the disease [24,25]. In this study, NPC patients showed the lowest percentage of positive family history of the disease among HNCA. Statistical analysis clarified that NPC group was associated significantly with the negative family history of the disease while other HNCA groups were associated significantly with the positive family history of the disease. This implies to the notion that the environmental risk factors most probably play more important role in the etiology of NPC than the familial genetic factors. Moreover, the positive family history group of NPC showed far lower levels of serum EBV IgG and IgA antibodies than negative family history group. These findings together referred to many points to discuss. First, NPC is most affected by environmental than genetic factors. Second, patients with genetically predisposed NPC are unlikely associated with EBV as a risk or predisposing factor. Third, EBV etiology of NPC patients is less likely affected by familial and inheritable susceptibility and it acts as an independent risk factor. Therefore, it was proposed that the familial carcinogenic background of NPC patients might not be so essential for it's etiology like in CL and HPC suggesting that the biological and environmental risk factors are dominant over the genetic factors for NPC development.

NPC is often difficult to be diagnosed because of the non-specific nature of its clinical symptoms and the difficulty in visualizing the nasopharynx [26]. Most of NPC tumors remain undiagnosed until metastasis to the cervical lymph nodes occurs, often without overt pathology at the primary site [27,28] which indicates the desperate need for an early diagnosis and early treatment strategies to improve survival. Early detection of NPC should improve cure rate and reduce morbidity and metastasis. Taking into account the high incidence of NPC in many regions of the world and its difficult early diagnosis, hence the determination of the correct high risk groups of NPC in every society seems invaluable and life saving. EBV serological screening assays might be one of the reliable means for the population screening of NPC.

## Conclusion

Taken together, NPC showed a particular profile of risk factors that deviate much from other HNCA groups. NPC was particularly affected and triggered/promoted by EBV, LE tumors, alcohol intake, smoking habit, male patients. Unlike other studied HNCA groups, NPC was not affected much by HNCA-positive family history. On the other hand, the risk factors influence the development of other HNCA patients were smoking, increased age, male patients for CL and female patients for HPC, positive family history, and SCC tumors. Interestingly, this study also showed that higher levels of serum EBV IgG and IgA antibodies in NPC patients were associated with HNCA-negative family history, regular smoker group of NPC, LE histology of NPC, and younger NPC patients. In contrast, EBV serum antibodies were not associated with regular alcohol consumers or the sex of NPC patients. Hence, smoking and LE histology in NPC group might act as dependent risk factors via reactivating EBV infection. In contrast, alcohol consumption was associated with SCC tumor of NPC rather than higher levels of serum EBV IgG antibodies indicating that alcohol consumption is an EBV-independent risk factor for the development of NPC mainly of SCC histology. In addition, EBV risk factor might hasten the incidence of NPC patients in younger age. This study clarified the central role of EBV in controlling other risk factors of NPC making this disease distinct in terms of risk factors profile and incidence distribution. In addition, it is thought that a screening test for measuring the serum level of EBV IgA and IgG antibodies in the correctly determined high risk groups of a population will help much establish the correct basis for the early diagnosis and proper treatment of NPC patients.

## Competing interests

The authors declare that they have no competing interests.

## Authors' contributions

ASA carried out the sampling, patients examination, Data sheet survey, and samples of blood and paraffin embedded sections. NA carried out the processing of the paraffin embedded sections and histological examination. RRH and FA carried out the ELISA assay for in vitro synthesis of cytokines and paper draft. KAA carried out the statistical design, statistical analysis, and the proofreading of the article language and integrity. All authors read and approved the final manuscript.

## Pre-publication history

The pre-publication history for this paper can be accessed here:



## References

[B1] Erkal HS, Serin M, Cakmak A (2001). Nasopharyngeal carcinomas: analysis of patient, tumor and treatment characteristics determining outcome. Radiother Oncol.

[B2] Cooper JS, Lee H, Torrey M, Hochster H (2000). Improved outcome secondary to concurrent chemoradiotherapy for advanced carcinoma of the nasopharynx: preliminary corroboration of the intergroup experience. Int J Radiat Oncol Biol Phys.

[B3] Burt RD, Vaughan TL, McKnight B (1992). Descriptive epidemiology and survival analysis of nasopharyngeal carcinoma in the United States. Int J Cancer.

[B4] Yu MC, Yuan JM (2002). Epidemiology of nasopharyngeal carcinoma. Semin Cancer Biol.

[B5] Busson P, Ooka T, Corbex M (2004). Nasopharyngeal carcinomas and Epstein-Barr virus: from epidemiology and detection to therapy. Med Sci (paris).

[B6] Ou SH, Zell JA, Ziogas A, Anton-Culver H (2007). Epidemiology of nasopharyngeal carcinoma in the United States: improved survival of Chinese patients within the keratinizing squamous cell carcinoma histology. Ann Oncol.

[B7] Ng WT, Choi CW, Lee MC, Chan SH, Yau TK, Lee AW (2008). Familial nasopharyngeal carcinoma in Hong Kong: epidemiology and implication in screening. Fam Cancer.

[B8] Akeshita H, Furukawa M, Fujieda S, Shoujaku H, Ookura T, Sakaguchi M, Ito H, Mineta H, Harada T, Matsuura H (1999). Epidemiological research into nasopharyngeal carcinoma in the Chubu region of Japan. Auris Nasus Larynx.

[B9] Iraqi cancer Board (2003). Annual Iraqi cancer registry bulletin.

[B10] Tang JG, Li Xuan, Cheng P (2004). Expression of matrix metalloproteinase-9 in nasopharyngeal carcinoma and association with Epstein-Barr virus infection. J Zhejiang Univ SCI.

[B11] Serraino D, Piselli P, Angeletti C, Scuderi M, Ippolito G, Capobianchi MR (2005). Infection with Epstein-Barr virus and cancer: an epidemiological review. J Biol Regul Homeost Agents.

[B12] Pattle SB, Farrell PJ (2006). The role of Epstein-Barr virus in cancer. Expert Opin Biol Ther.

[B13] Baumforth KR, Young LS, Flavell KJ, Constandinou C, Murray PG (1999). The Epstein-Barr virus and its association with human cancers. Mol Pathol.

[B14] UICC (1978). TNM Classification of Malignant Tumors.

[B15] Wakabayashi I, Kobaba-Wakabayashi R, Masuda H (2002). Relation of drinking alcohol to atherosclerotic risk in type 2 diabetes. Diabetes Care.

[B16] Schuurs A, Weeman V (1977). Enzyme innunoassay. Clin Chem Acta.

[B17] Cheng WM, Chan KH, Chen HL, Luo RX, Ng SP, Luk W, Zheng BJ, Ji MF, Liang JS, Sham JS (2002). Assessing the risk of nasopharyngeal carcinoma on the basis of EBV antibody spectrum. Int J Cancer.

[B18] Koshiol J, Qiao YL, Mark SD, Dawsey SM, Abnet CC, Kamangar F, Lennette ET, Dong ZW, Taylor PR (2007). Epstein-Barr virus serology and gastric cancer incidence and survival. Br J Cancer.

[B19] Fachiroh J, Schouten T, Hariwiyanto B, Paramita DK, Harijadi A, Haryana SM, Ng MH, Middeldorp JM (2004). Molecular diversity of Epstein-Barr virus IgG and IgA antibody responses in nasopharyngeal carcinoma: a comparison of Indonesian, Chinese, and European subjects. J Infect Dis.

[B20] Chan KH, Gu YL, Ng F, Ng PS, Seto WH, Sham JS, Chua D, Wei W, Chen YL, Luk W (2003). EBV specific antibody-based and DNA-based assays in serologic diagnosis of nasopharyngeal carcinoma. Int J Cancer.

[B21] Sankaranarayanan R, Masuyer E, Swaminathan R, Ferlay J, Whelan S (1998). Head and neck cancer: a global perspective on epidemiology and prognosis. Anticancer Res.

[B22] Young LS, Murray PG (2003). Epstein-Barr virus and oncogenesis: from latent genes to tumours. Oncogene.

[B23] Berrino F, Gatta G (1998). Variation in survival of patients with head and neck cancer in Europe by the site of origin of the tumours. Eur J Cancer.

[B24] Terrell JE, Nanavati K, Esclamado RM, Bradford CR, Wolf GT (1999). Health impact of head and neck cancer. Otolaryngol-Head-Neck-Surg.

[B25] Chang ET, Adami HO (2006). The enigmatic epidemiology of nasopharyngeal carcinoma. Cancer Epidemiol Biomarkers Prev.

[B26] ong YS, Sham JS, Ng MH, Ou XT, Guo YQ, Zheng SA, Liang JS, Qiu H (1992). Immunoglobulin A against viral capsid antigen of Epstein-Barr virus and indirect mirror examination of the nasopharynx in the detection of asymptomatic nasopharyngeal carcinoma. Cancer.

[B27] Feinmesser R, Miyazaki I, Cheung R, Freeman JL, Noyek AM, Dosch HM (1992). Diagnosis of nasopharyngeal carcinoma by DNA amplification of tissue obtained by fine-needle aspiration. N Engl J Med.

[B28] Zheng H, Li LL, Hu DS, Deng XY, Cao Y (2007). Role of Epstein-Barr virus encoded latent membrane protein 1 in the carcinogenesis of nasopharyngeal carcinoma. Cell Mol Immunol.

